# Feasibility study of a novel acquisition technique of cardiac cine magnetic resonance imaging in patients with atrial fibrillatio

**DOI:** 10.1186/1532-429X-17-S1-T12

**Published:** 2015-02-03

**Authors:** Jian Cao, Yining Wang, Lingyan Kong, Lu Lin, Yan Yi, Jing An, Tianjing Zhang, Zhengyu Jin

**Affiliations:** 1 Radiology, Peking Union Medical College Hospital, Beijing, China; 2 MR Collaborations NE Asia, Siemens Healthcare, Beijing, China

## Background

For patients with arrhythmia, conventional segmented steady-state free precession sequence (CINE-SSFP) methods may result in poor image quality. Real-time cine could avoid those limitations, however poor spatial and temporal resolutions of conventional sequences have prevented its routine application. Recently a newly developed compressed sensing (CS) cine shows the ability to provide both spatial and temporal resolution in real time. The purpose of this study is to assess its performance in patients with atrial fibrillation.

## Methods

CMR was performed at 3.0T system (MAGNETOM Skyra, Siemens Healthcare, Erlangen, Germany). The examinations included the following sequences (Table 1): (1), conventional segmented SSFP (iPAT3) (GRAPPA accel factor 3, temporal resolution, 45.6 ms;voxel size, 1.6 x 1.6 x 8.0mm3); (2), conventional real-time (TPAT3) (TGRAPPA accel factor 3, temporal resoluion49.9 ms; voxel size 2.8×2.8×8.0mm3); (3), segmented CS (SPARSE11.5) (accel factor 11.5, temporal resolution, 41.7 ms; voxel size 2.0×2.0×8.0 mm3); (4),real-time CS (SPARSE9.3) (accel factor 9.3, TR, 17 ms; voxel size, 2.0×2.0×8.0mm3 ). Stacks of short-axis (SAX) cines were acquired covering both ventricles. For every subject, three SAX slices (base, mild and apex) of each sequence were selected and reviewed in the random order. Image quality scoring was performed by two experts using 5-point score (1-5 from poor to good).

**Table 1:** Comparisons of scan time and image quality

Sequence	Scan time (sec/slice)	Overall image quality (1-5 from poor to good)
iPAT3	6.0±0.0	3.7±0.5 (base)
		
		3.8±1.0 (mild)
		
		3.8±0.7 (apex)

TPAT3	0.8±0.1	3.6±0.5(base)
		
		3.6±0.5 (mild)
		
		3.6±0.5(apex)

SPARSE11.5	1.6±0.2	4.1±0.8(base)
		
		4.2±0.4 (mild)
		
		4.2±0.8(apex)

SPARSE9.3	3.1±0.5	4.2±0.4(base)
		
		4.6±0.5 (mild)
		
		4.8±0.4(apex)

## Results

**Figure 1:**
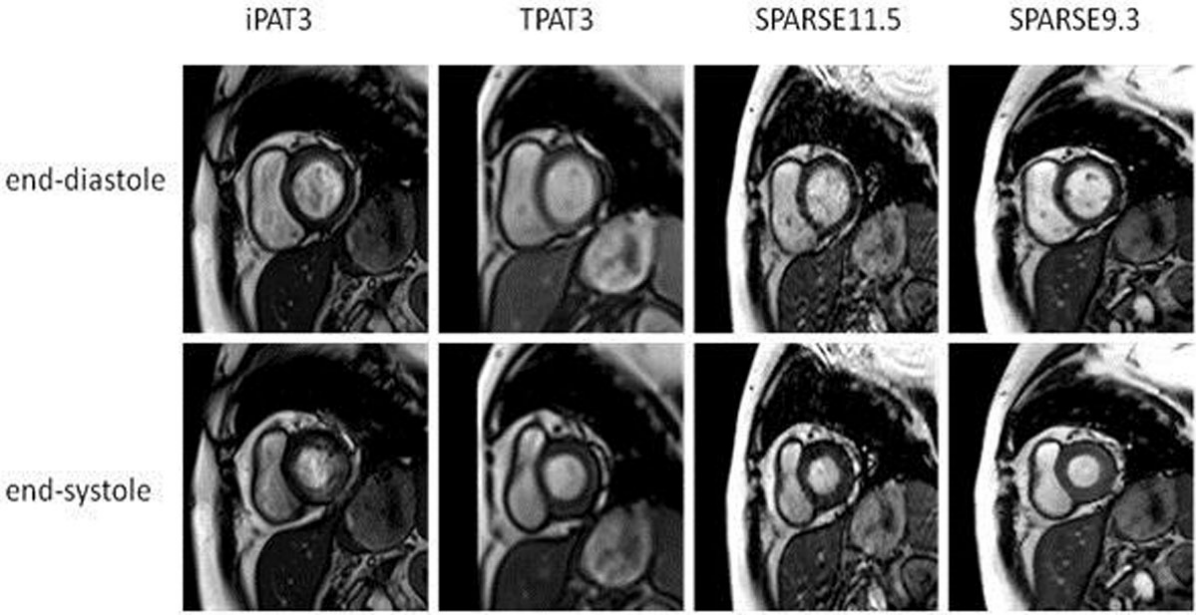
Visual comparison of the conventional segmented (iPAT3),conventional real-time (TPAT3), segmented CS (SPARSE11.5) and real-time CS - (SPARSE9.3) CMR in a 73 year-old female patient (base-segment).

CMR examinations of 6 patients (46±18 years, 1 male) with AF were completed successfully. Short axis slice acquisition times were shorter for TPAT3 (0.8± 0.1 s) than iPAT3 (6.0± 0.0 s), SPARSE11.5 (1.6± 0.2 s) and SPARSE9.3 (3.1± 0.5 s)(Table 1). But on overall image quality, the SPARSE9.3 technique was best, which was superior to iPAT3, TPAT3 and SPARSE11.5(Table 1), neither standard segmented SSFP nor standard real-time sequence could have diagnostic quality. And the image quality of base-segments were worse than mild and apex segments because of flow artifacts (Fig 1)

## Conclusions

Our small sample research indicates that for patients with arrhythmia, the conventional SSFP and real-time sequences could not fulfill the clinical requirements. With the application of CS cine sequence, we could acquire high-quality cardiac cine images which greatly reduce the scan time. In future, it could be applied in more clinical patients.

## Funding

N/A.

